# Supplementary material for: Mutations in PCYT2 disrupt etherlipid biosynthesis and cause a complex hereditary spastic paraplegia
Source: Brain. 2019 Oct 22;142(11):3382–97. doi: 10.1093/brain/awz291 (PMC6821184; doi:10.1093/brain/awz291)
Supplement: awz291_Supplementary_Data [file awz291_supplementary_data.zip › awz291-Suppl_data/Supplementary_Data4.zip!/Supplementary information 3 - fibroblast lipidomics.zip!/Fibroblast lipidomics/Controle vs Patient2/Statistical plots and data/LPC[O] species/Boxplots all.pdf]

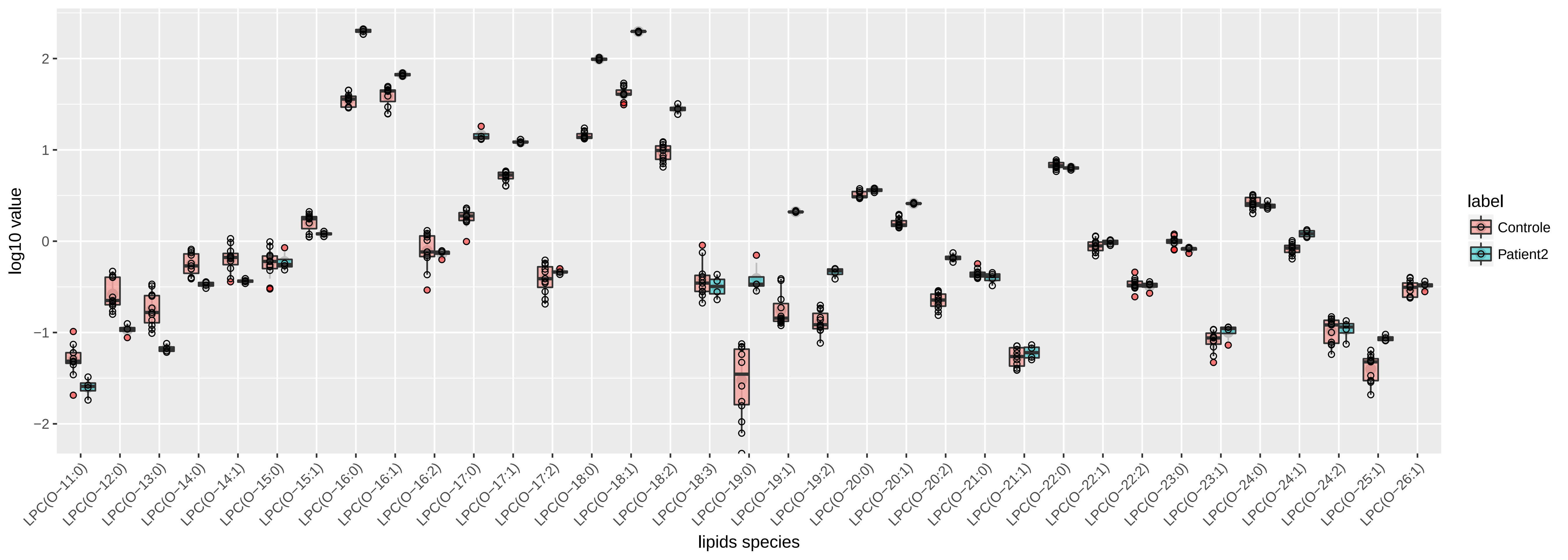

log10 value
2
1
0
−1
−2
LPC(O−11:0)
LPC(O−12:0)
LPC(O−13:0)
LPC(O−14:0)
LPC(O−14:1)
LPC(O−15:0)
LPC(O−15:1)
LPC(O−16:0)
LPC(O−16:1)
LPC(O−16:2)
LPC(O−17:0)
LPC(O−17:1)
LPC(O−17:2)
LPC(O−18:0)
LPC(O−18:1)
LPC(O−18:2)
LPC(O−18:3)
LPC(O−19:0)
LPC(O−19:1)
LPC(O−19:2)
LPC(O−20:0)
LPC(O−20:1)
LPC(O−20:2)
LPC(O−21:0)
LPC(O−21:1)
LPC(O−22:0)
LPC(O−22:1)
LPC(O−22:2)
LPC(O−23:0)
LPC(O−23:1)
LPC(O−24:0)
LPC(O−24:1)
LPC(O−24:2)
LPC(O−25:1)
LPC(O−26:1)
lipids species
label
Controle
Patient2
